# Attosecond stable dispersion-free delay line for easy ultrafast metrology

**DOI:** 10.1038/s41598-022-12348-5

**Published:** 2022-05-20

**Authors:** Akansha Tyagi, Mehra S. Sidhu, Ankur Mandal, Sanjay Kapoor, Sunil Dahiya, Jan M. Rost, Thomas Pfeifer, Kamal P. Singh

**Affiliations:** 1 Department of Physical Sciences, Indian Institute of Science Education and Research Mohali, Sector-81, Manauli, Mohali, 140306 India; 2 Max Planck Institute for Physics of Complex Systems, Nöthnitzer Straße 38, 01187 Dresden, Germany; 3 Max Planck Institute for Nuclear Physics, D-69117 Heidelberg, Germany

**Keywords:** Applied optics, Lasers, LEDs and light sources, Optical physics, Optical techniques

## Abstract

We demonstrate a dispersion-free wavefront splitting attosecond resolved interferometric delay line for easy ultrafast metrology of broadband femtosecond pulses. Using a pair of knife-edge prisms, we symmetrically split and later recombine the two wavefronts with a few tens of attosecond resolution and stability and employ a single-pixel analysis of interference fringes with good contrast using a phone camera without any iris or nonlinear detector. Our all-reflective delay line is theoretically analyzed and experimentally validated by measuring 1st and 2nd order autocorrelations and the SHG-FROG trace of a NIR femtosecond pulse. Our setup is compact, offers attosecond stability with flexibility for independent beam-shaping of the two arms. Furthermore, we suggest that our compact and in-line setup can be employed for attosecond resolved pump-probe experiments of matter with few-cycle pulses.

## Introduction

Optical delay lines are key tools in ultrafast science and technology with several applications from femtosecond metrology to attosecond pump-probe spectroscopy of matter^1–7^ . Most delay lines are based on either amplitude division or wavefront division of an input beam. The conventional Michelson or Mach-Zehnder setups employ transmission through mm-thick glass beam splitter for splitting (combining) a femtosecond (fs) pulse in the infrared (IR) or near UV, thereby causing unwanted broadening and material-dispersion effects on the pulse^8,9^. Careful fabrication of dispersion balanced beam splitter has been shown to enable the measurement of short fs-pulses^10,11^. Grating-based beam splitters have also been employed in delay lines, however, with compromised energy throughput and limited operating wavelengths^12,13^. Quasi-dispersion-free transmission grating and ultrathin delay lines minimize dispersion effects on pulses and achieve attosecond stability over tens of fs delay range^14–16^.

All reflective delay lines are ideal to avoid pulse distortion caused by transmission of a short pulse through a medium. Several designs have been well established where wavefront division is achieved by a bisected split mirror using plane, spherical, parabolic, toroidal, and double-comb mirror^7,17–20^ in oblique as well as grazing incidence angle geometry^21^. Due to the grazing incidence of the input beam, the focus drifts away as one of the split mirrors is linearly displaced to control the delay, which limits the useful delay range to a few tens of fs. Moreover, in grazing incidence split-and-focusing mirrors, it is difficult to achieve spatio-temporal overlap of foci on the detector, which reduces the fringe contrast^22–24^. Moreover, such geometries have limited freedom to independently control intensity, polarization and spectrum of individual split wavefronts. The present work addresses some of these above mentioned difficulties by demonstrating a knife-edge prism based all-reflective attosecond delay line.

An essential application of all-reflective delay lines is for complete characterization of broadband femtosecond pulses. Generally, the two spatially overlapped and time-delayed pulses are coupled into ultrathin nonlinear crystal to generate an output 2-photon or 3-photon autocorrelation signal which is sensitively detected using avalanche photodiodes^25,26^. To improve the contrast of autocorrelation traces, a pin-hole having fine position controls is required^27^ which makes the overall alignment and pulse characterization a tedious task. One may wonder how to design a dispersion-free compact delay line offering attosecond stability for easy ultrafast metrology.

Here, we introduce a knife-edge prism mirror pair based delay line for ultrafast metrology with attosecond resolution and stability. The wavefront of input fs-beam is symmetrically split and recombined via sharp-edged right-angle prism mirrors, thereby separating the two arms while avoiding dispersion and pulse broadening effects and maintaining the spatial overlap in delayed pulses over the entire delay range of > 500 fs. The working of delay line is theoretically analyzed and experimentally validated by easy temporal characterization of near-IR femtosecond pulses. We captured 1st and 2nd order interference patterns with a (handheld) phone-camera and introduce a single-pixel analysis to record high-resolution autocorrelation without any pin-holes and sensitive detectors. Furthermore, the setup also yields reliable measurement of Second harmonic generation -frequency-resolved optical gating (SHG-FROG) traces.

## Results

### Experimental setup

**Figure 1:**
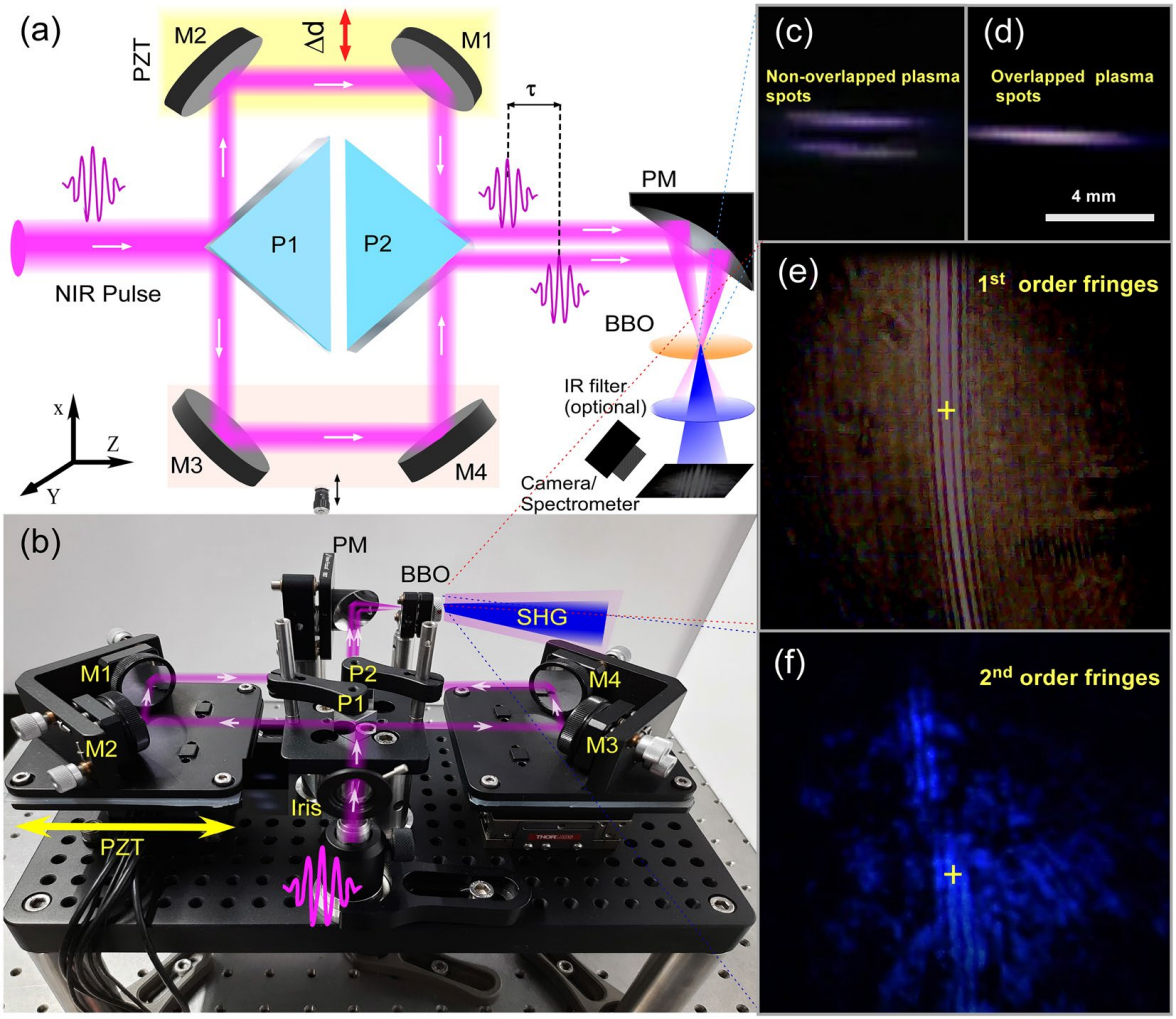
(**a**) Schematics of all-reflective delay line. M1–M4 : broadband mirrors (650–1100 nm), P1 and P2: Ag coated knife-edge prisms for beam splitting and recombination, PM: Parabolic mirror (f = 25.4 mm), M1 and M2 are placed on the piezo stage. (**b**) Picture of all-reflective delay line setup. (**c**) Two non-overlapping micro-plasma spots from individual arms. (**d**) Overlapped microplasma spots (**e**) 1st-order fringes corresponding to 800 nm on screen. (**f**) 2nd-order blue fringes generated by BBO. A spectrometer was used for FROG measurements.

The schematic diagram of our all-reflective delay line is shown in Fig. 1a along with the actual setup in Fig. 1b. Two identical knife-edge right-angle prisms P1 and P2 serve as a wavefront splitter and beam combiner of the input Gaussian beam, respectively. The sharpness of the knife-edges were around 20 μm (Supplementary Figs. S1–S2). The flatness of silver-coated surface was $\lambda /8$ at 632 nm and the reflectivity $R_{avg} >96\%$ in broad wavelength range covering visible to IR (450 nm–20 μm). The delay line was assembled on a small rigid base that allowed high mechanical stability yet retaining independent controls for both arms.

To precisely align the two split wavefronts in the target focal volume and to optimize the beam crossing angle, we made use of two static diffraction patterns, each produced by the knife-edge of the prism P1. By overlapping their 1st order fringes, a dynamic interference pattern was formed when both the pulses had good temporal overlap. This central fringe pattern was easily captured on the screen placed near or far from the focus using a phone-camera (20 MP, 25 fps). By aligning the fringes nearly perpendicular to the (horizontal) plane of the delay line, and by controlling the fringe spacing we precisely maintained the collinearity and wavefront tilt to obtain high-contrast, straight line fringes with high visibility.

After recombination of the two wavefront-split beams by the prism P2, these propagate collinearly and are focused by an off-axis parabolic mirror ($f=25.4$ mm). For peak intensity $\sim100$ GW/cm^2^, we observe two micro-plasma, each created by a breakdown of air by the split femtosecond pulses. These micro-plasma spots were readily observed with a webcam and were made to symmetrically overlap on each other by means of adjusting the mirrors (M1-M4) in the two independent arms, leading to an enhanced plasma brightness (Fig. 1c–d). The spatial overlap of the two microplasma spots along with optimization of the contrast, tilt and spacing of the interference fringes on the screen provided a unique and repeatable method to precisely align our delay line over its entire delay range.

Figure 1e shows the first-order fringes with 800 nm pulses as observed on the screen placed after the focus of the parabolic mirror at 5 cm. In order to obtain 2nd order interference fringes, we inserted a 25 $\mu$m BBO $(\beta$–BaB_2_0_4_) crystal (Type-1 phase matched). The intensity of the incident beam was reduced below the damage threshold of the BBO crystal using the crossed polarizer placed before the delay line. Figure 1f shows typical blue colored fringes captured through a IR blocking (short wavelength pass) filter. The fringes were video-recorded at 25 fps and stored for further analysis.

For generating field-autocorrelation signals (interferogram), we tracked single-pixel intensity of the straight-line fringe pattern frame-by-frame, at a position marked by a cross-hair in Fig. 1e. This unique approach did not require any precision pin-hole and was free from tedious detector alignment with added advantage of being cost effective as common phone-camera can be used for measuring both the first-order (800 nm) and second-order (SHG 400 nm) fringe pattern of a broadband NIR fs pulse.

### Theoretical analysis

To further elaborate the working of our delay line, we used Fraunhofer diffraction theory^28^ and numerically simulated the autocorrelation signal following a previously established approach^22,27,29^. Briefly, the two split beams having equal peak electric field were symmetrically combined in the focal plane leading to straight-line fringes at the center of the overlapping region. The spatial profile of both split beams at focus having angular frequency $\omega$ and initial beam spot size *w*(*z*) is given as,

1 $$U_{{1,2}} (x,y,\omega ) = \frac{i}{\omega }\frac{{cf}}{{w_{0}^{2} }}exp\left( { - \frac{{x^{2} + y^{2} }}{{w_{0}^{2} }}} \right)\left[ {1 \pm i\frac{{2x}}{{\sqrt \pi w_{0} }}\left| F \right|\left[ {\frac{1}{2},\frac{3}{2},(\frac{x}{{w_{0} }})^{2} } \right]} \right]{\text{ }}$$

where *x* and *y* are the coordinates of the observation plane, $\shortmid F \shortmid$ is the confluent hypergeometric function, f is the focal length of the mirror and $w_{0}$ is the beam waist at focus ($w_{0}=\lambda f/\pi w$).

2 $$\begin{aligned} E_{1,2}(x,y,t)=\int _{-\infty }^{\infty }E(\omega )U_{1,2}(x,y,\omega )exp(-i\omega t)d\omega \end{aligned}$$

$E(\omega )$ is laser electric field, $E_{1}(x,y,t)$, $E_{2}(x,y,t-\tau )$ are the electric fields of first and second delayed split beams at focus and $\tau$ is the time delay between the two split beams.

By changing the relative delay between the two spatially split beams the first-order interferogram is obtained as

3 $$\begin{aligned} I_{\omega }[\tau ]=1+Re[\int \int \int E_{1}(x,y,t)E_{2}^{*}(x,y,t-\tau ) exp(-i\omega \tau ) dxdydt] \end{aligned}$$

Second-order normalized interferometric autocorrelation or frequency-resolved auto-correlation (FRAC) signal is given by^11^

4 $$\begin{aligned} I_{2\omega }[\tau ]=1+2[A_{0}(\tau )+Re[A_{1}(\tau )exp(-i\omega \tau )]+Re[A_{2}(\tau ) exp(-i2\omega \tau )] \end{aligned}$$

where,

5 $$\begin{aligned} A_{0}(\tau )=\int \int \int I_{1}(x,y,t)I_{2}(x,y,t-\tau ) dxdydt \end{aligned}$$

6 $$\begin{aligned} A_{1}(\tau )=\int \int \int [I_{1}(x,y,t)+I_{2}(x,y,t-\tau )]E_{1}(x,y,t) E_{2}^{*}(x,y,t-\tau )dxdydt \end{aligned}$$

7 $$\begin{aligned} A_{2}(\tau )=\int \int \int E_{1}^{2}(x,y,t)E_{2}^{*2}(x,y,t-\tau ) dxdydt \end{aligned}$$

where, $I_{1,2}(x,y,t) \sim |E_{1,2}(x,y,t)|^{2}\delta A$. The autocorrelation signal is detected at a single-pixel of area ($\delta A$=$\delta x \delta y$) and centered at $(x_{p}, y_{p})$ over the complete time-delay range. In FRAC equation (Eq. 4), $A_{0}(\tau )$ shows the intensity autocorrelation envelope, $A_{1}(\tau )$ and $A_{2}(\tau )$ are linear interferogram and second harmonic field, respectively which are separated in frequency space. The Fourier transform of Eq. (3) provides the spectral information. One can further obtain the phase information of the fundamental pulse using $A_{0}(\tau )$, $A_{1}(\tau )$ and $A_{2}(\tau )$^30,31^. Alternatively, the SHG-FROG can also be used for non-ambiguous phase retrieval^32^.

### Autocorrelation and FROG measurements

**Figure 2:**
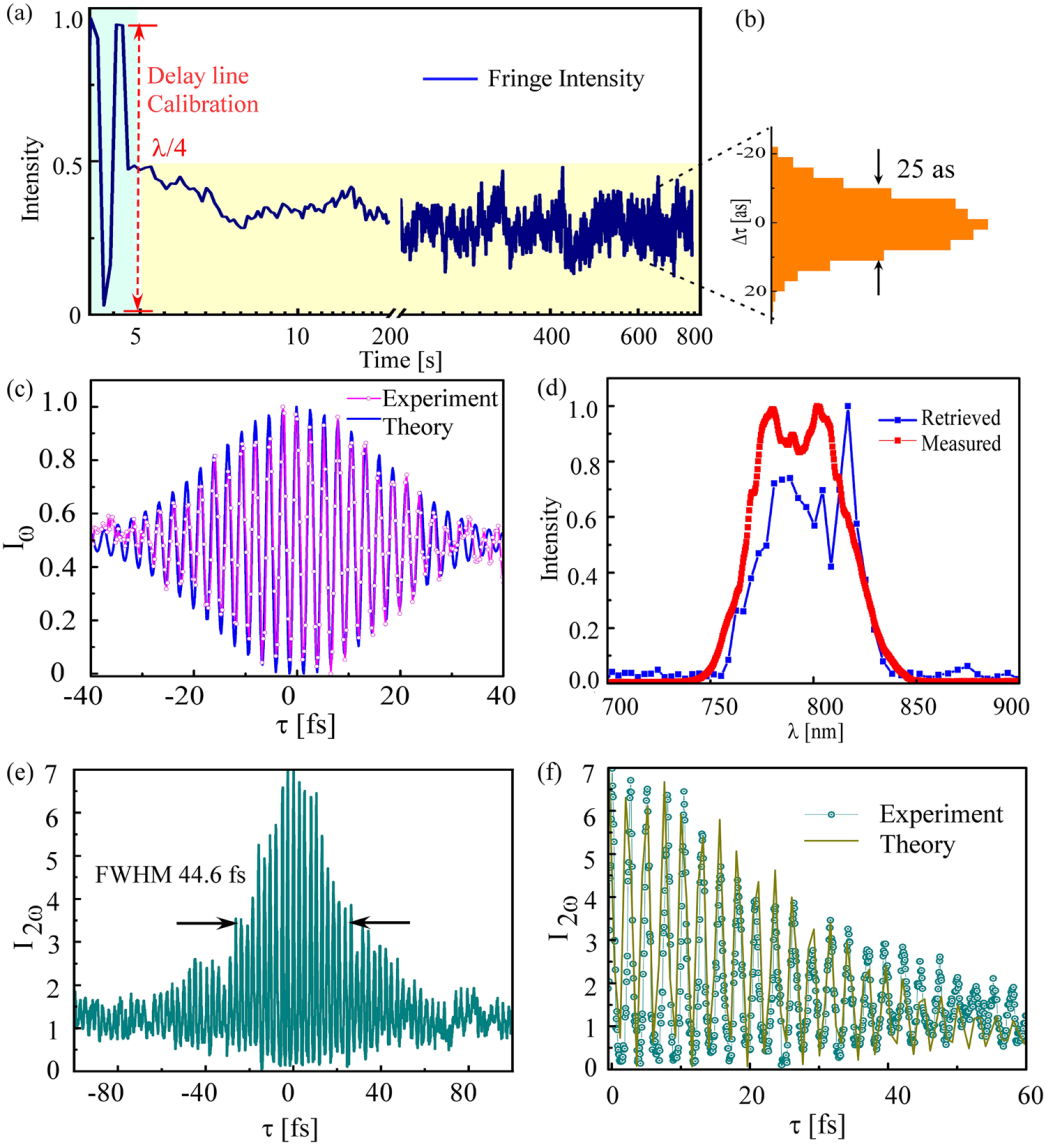
(**a**) Calibration of delay line through interferometric fringes and stability analysis of all-reflective delay line measured with a He-Ne laser $(\lambda\,=\,632$ nm) over time. (**b**) Normalized histogram of path delay fluctuation. (**c**) Experimental and theoretical linear autocorrelation signals. (**d**) Measured (red) and Retrieved (blue) spectra through first-order interferogram. (**e**) Experimental SHG non-linear autocorrelation signal using BBO. (**f**) Experimental and theoretical autocorrelation traces based on Fraunhofer diffraction theory for split profile of the beam for positive delay range (0–60 fs).

To demonstrate the high stability of our delay line for ultrafast measurements we performed a stability analysis. First, to isolate the delay line from fluctuations due to air-flow or mechanical vibrations all the components were firmly mounted on a 1 inch thick monolithic platform ($304.8\times 152.4\times 22$ mm^3^) which was further enclosed in an acrylic box with two large enough openings to couple the input and output beams. The stability analysis and calibration of the present all-reflective delay line is performed using He–Ne laser (5 mW, $\lambda = 632$ nm) as shown in Fig. 2a by tracking a single fringe over time while the PZT is fixed at one position in a closed loop operation. The initial fringes (blue shaded part) are used for calibration of delay line which corresponds to $(\lambda /4)$ from bright to dark fringe. Figure 2b shows the histogram for path-length fluctuation, the standard deviation was about 25 as $(\lambda /90)$ over 800 seconds without any active stabilization.

Figure 2c shows the measured and theoretical interferogram using Eq. (3) of the fundamental pulse (800 nm) as a function of variable time delay between two replica beams by tracking the first-order fringes. The obtained first-order interferogram is useful in providing pulse spectral information but unable to provide pulse temporal parameters. Figure 2d shows the recovered spectrum from the recorded interferogram and the measured spectrum of the fundamental pulse with an IR spectrometer (USB2000+, Ocean optics) covering the wavelength range 515–1100 nm which shows a fair agreement. Figure 2e shows the second-order fringe-resolved autocorrelation (FRAC) signal obtained through blue fringes as shown in Fig. 1f after BBO. The signal is obtained with a constant delay step of 200 as and acquisition time of 100 ms and total measurement time of 100 s. The measured second-order autocorrelation trace shows contrast  7:1 which is slightly lower as compared to its ideal value of 8:1 expected for the case of whole beam interferometer. Previously reported split-mirror autocorrelators approached a low fringe contrast of  5:1^22^, which was further increased to  7.96:1^27^ by introducing a pin hole before detector in the similar set up. In our case of single pixel analysis, the signal-to-noise ratio is sensitive to thermal noise of the camera, stray light and laser pointing fluctuation. The pulse width measured with autocorrelation trace with all-reflective delay line shows FWHM 44.6 fs, supports 31 fs pulse duration having deconvolution factor of 1.41 assuming Gaussian temporal profile of the fundamental pulse. The measured autocorrelation signal is compared with simulated FRAC signal using Eq. (4) for second-order autocorrelation considering a split beam spatial profile is shown in Fig. 2f. It shows a good agreement between experimental and simulated autocorrelation signal. With the present autocorrelator design no further correction factor due to beam splitters and other optics is required in measuring the pulse via FRAC signal unlike in conventional autocorrelators based on transmissive-optics beam splitter^10^.

**Figure 3:**
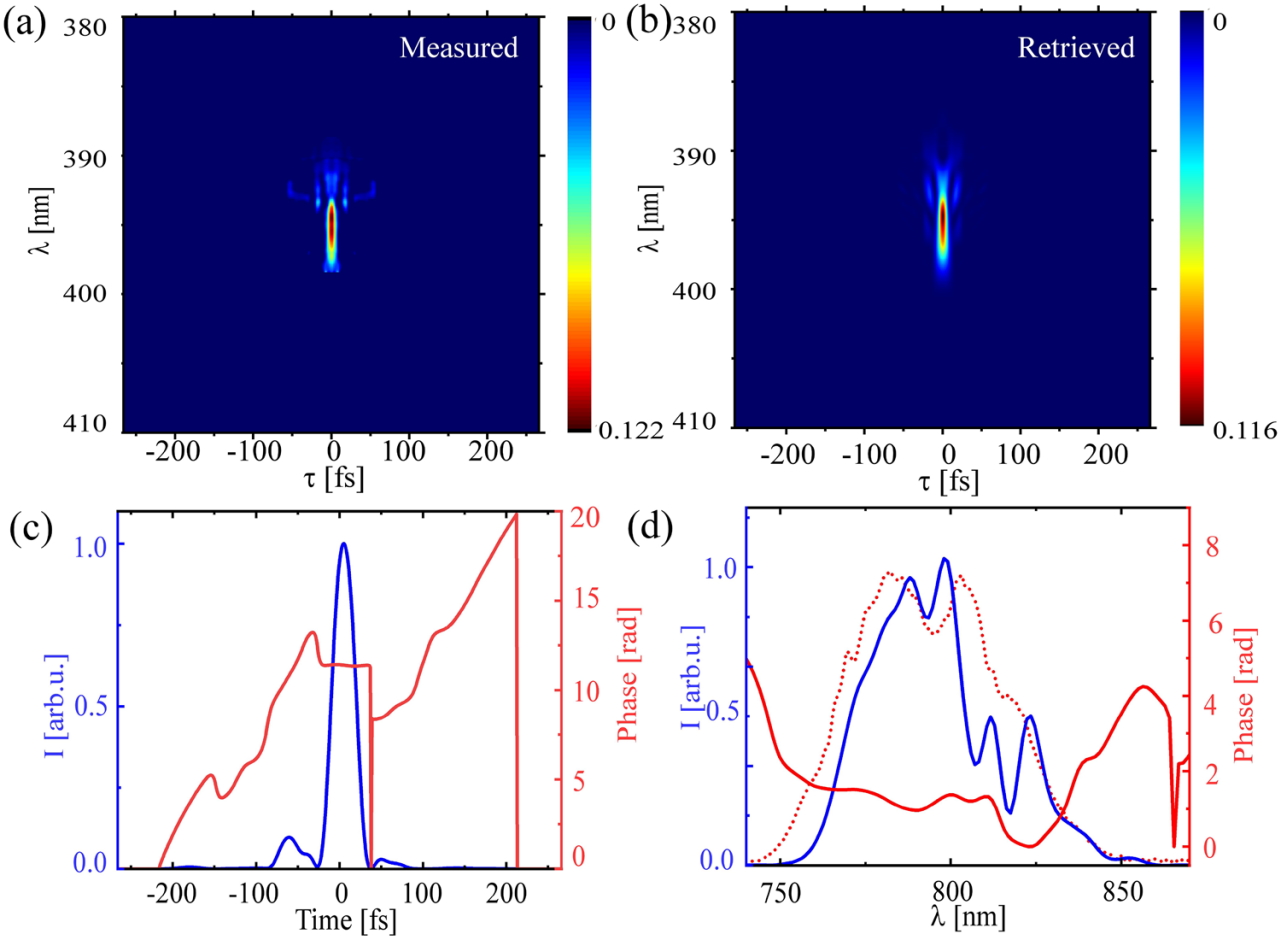
FROG measurement results (**a**) Measured SHG-FROG trace with all-reflective delay line. (**b**) Retrieved FROG trace. (**c**) Retrieved temporal pulse intensity (left y-axis) and phase (right y-axis). (**d**) Retrieved spectral intensity (left y-axis) and phase(right y-axis). Dotted red line shows the measured spectrum, same as in Fig. 2d, for comparison.

For complete reconstruction, we have obtained the FROG trace of our pulse by recording SHG spectra at each time delay position (delay step of 200 as, 50 ms acquisition time, total measurement time of 100 s) over a delay scan of $\tau =$ − 266 to 266 fs. The FROG trace was obtained by low-pass frequency filtering of measured IFROG (Interferometric frequency resolved optical gating) trace by applying super-gaussian filter about DC component in the Fourier transform (along delay axis) of the IFROG trace. This DC part contains the SHG-FROG and a constant background term^33^. The obtained standard SHG-FROG trace is shown in Fig. 3a. Figure 3b shows the retrieved FROG trace that qualitatively matches with the input one. The retrieval of the experimental FROG trace is performed by resampling the trace to an $512\times 512$ array via the open source software Femtosoft (Femtosoft, Ver 3.2.4) using principal component generalized projection algorithm (PCGPA)^32^ and minimization algorithm^34^ which provides the retrieved trace with minimum error. The retrieved intensity and temporal phase are shown in Fig. 3(c) and the corresponding wavelength spectrum and spectral phase profile are shown in Fig. 3d. The retrieved spectrum shows a reasonable agreement with the measured spectrum, plotted as dotted red line in Fig. 3d.

## Conclusions

In conclusion, we demonstrate an all-reflective two-arm attosecond delay line based on wavefront division (recombination) using right-angle prism mirrors for dispersion-free ultrafast metrology. The delay line is experimentally validated by measuring the first- and second-order autocorrelations generated by single-pixel analysis of interference pattern in agreement with theory and retrieval of the SHG-FROG without any iris or sensitive detector. The unique geometry of our delay line allows easy alignment for spatio-temporal overlap of a short femtosecond pulse without astigmatism and chromatic aberration over its entire delay range. Additionally, independent control of each split beam (power, polarization, angular momentum and spectrum) is possible^35–37^.

We envision that the present dispersionless compact attosecond delay line will find applications in attosecond resolved IR-IR or IR-VIS pump-probe experiments of matter, precision metrology and optical gating with few-cycle pulses^38–40^. We think that the present delay line can be used for XUV-IR attosecond experiments. For example, one can split the input pulse asymmetricaly such that the strong arm is used for high harmonic generation while the weak one can serve as a time-delayed collinear probe^15^. Furthermore, using vertically stacked prisms pairs and mirrors, a quadrant splitting of the input wavefront should be possible for multidimensional IR-IR and XUV-IR ultrafast experiments^41^.

## Methods

The experiments were carried out with a linearly polarized femtosecond beam of $1/e^2$ diameter 12 mm at a central wavelength of 800 nm having 0.5–1 mJ energy per pulse at 1 kHz repetition rate. The input beam was symmetrically split in two equal parts by the P1 prism, which were made to retro-reflect through a pair of static broadband mirrors (650–1100 nm), in each arm of the interferometer, and recombined by the prism P2.

The delay line has a coarse (manual) and fine (piezoelectric) time delay control. The coarse adjustment was manually performed to roughly overlap the two IR pulses in time using a micro-positioner (step-size 10 μm) on which the pair of mirror (M3 and M4) were mounted. The temporal overlap of the two pulses was manifested by appearance of dynamic straight-line interference fringes. The fine as-resolved movement was achieved by the other arm (M1 and M2) using a piezo-based nano-positioner (NPXYZ100, Newport) over a maximum possible delay range of 532 fs (corresponding to 80 μm maximum piezo displacement with a 4 nm step resolution, i.e., 26.6 as delay step). The dwell-time (acquisition time) per delay step was adjusted from 0.1 to 100 s as desired by the experiment. Furthermore, our design allows extension of the maximum time-delay range by replacing the existing piezo-stage with another one allowing longer time delay range up to a few picoseconds.

## Supplementary Information

Supplementary Information.

## Data Availability

Data/codes used for this study can be obtained from corresponding author on reasonable request.
