# Supplementary material for: Attosecond stable dispersion-free delay line for easy ultrafast metrology
Source: Sci Rep. 2022 May 20;12:8525. doi: 10.1038/s41598-022-12348-5 (PMC9122952; doi:10.1038/s41598-022-12348-5)
Supplement: Supplementary file 1 — Supplementary Information. [file 41598_2022_12348_MOESM1_ESM.pdf]

Supplementary: Attosecond stable dispersion-free delay line for easy ultrafast metrology

Akansha Tyagi[1], Mehar S. Sidhu[1], Ankur Mandal[1], Sanjay Kapoor[1], Sunil Dahiya[1], Jan M. Rost[2], Thomas Pfeifer[3], Kamal P. Singh[1]

Department of Physical Sciences, Indian Institute of Science Education and Research Mohali, Sector 81, Manauli 140306, India.

Max Planck Institute for Physics of Complex Systems, Dresden, Germany

Max Planck Institute for Nuclear Physics, Heidelberg, Germany

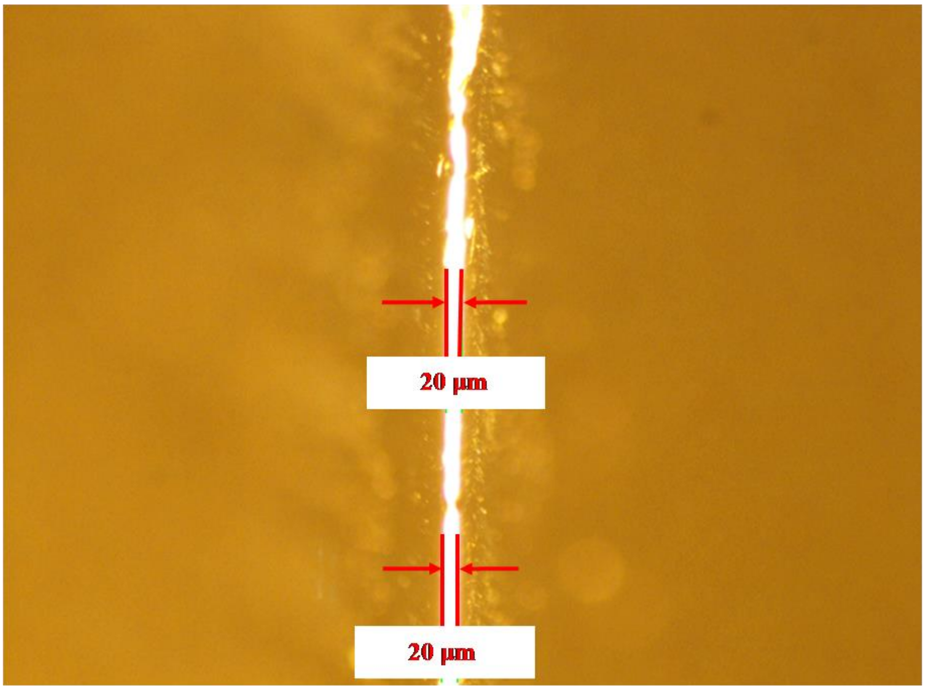

Figure S1: Optical microscopic image of reflective beam splitting knife edge prism used in all reflective delay line, showing knife edge sharpness of the order of ≈ 20 µm

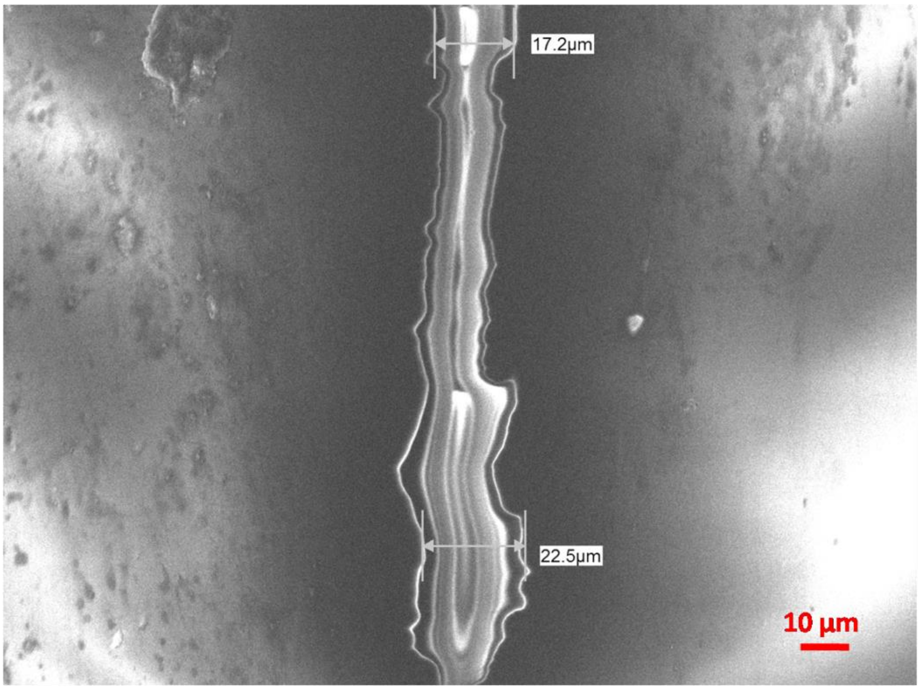

Figure S2: Electron microscopic image of reflective beam splitting knife edge prism
